# 

**Published:** 1874-05

**Authors:** 


### CONTENTS.

### COMMUNICATIONS.

Abnormal Mucous Membrane ..... ..... 185
Revelations of the Microscope ..... 193
Hernia of the Dental Pulp ..... 198
Address to the graduates of the Ohio College of Dental Surgery ..... 203
Reply on behalf of the class of 1874 ..... 211

### CORRESPONDENCE.

Letter from China ..... 214
Revival in Dental Journalism ..... 217

### EDITORIAL.

To our Contributors ..... 219
To the Profession ..... 220
Associations ..... 223
History of Dentistry ..... 225
Illinois State Dental Society ..... 226

